# Shimmering emerging adulthood: in search of the invariant IDEA model for collectivistic countries

**DOI:** 10.3389/fpsyg.2024.1349375

**Published:** 2024-04-08

**Authors:** Victoria G. Yerofeyeva, Pai Wang, Yisheng Yang, Astghik K. Serobyan, Ani K. Grigoryan, Sofya K. Nartova-Bochaver

**Affiliations:** ^1^School of Psychology, HSE University, Moscow, Russia; ^2^School of Psychology, Inner Mongolia Normal University, Hohhot, Inner Mongolia Autonomous Region, China; ^3^Department of Personality Psychology, Faculty of Philosophy and Psychology, Yerevan State University, Yerevan, Armenia

**Keywords:** emerging adulthood, IDEA, instability, self-focus, feeling in-between, experiments, identity exploration, cross-cultural research

## Abstract

Emerging adulthood is the youth trajectory characterized by self-focus, identity exploration, feeling between adolescence and adulthood, instability, and experimentation. This trajectory was first identified in industrialized individualistic countries with gender equality and technological progress. To measure transition to adulthood, the Inventory of the Dimensions of Emerging Adulthood (*IDEA*) was created. Although emerging adulthood is considered universal, adaptations of the questionnaire across the 12 countries show different patterns, and its cross-cultural invariance has been underinvestigated. This study tests *IDEA* in three collectivistic countries – Armenia, China, and Russia. The sample consisted of 868 students (total male – 152, total female – 716) aged 18 to 29 years old. We tested the questionnaire separately in the three countries to check that this model fits, but we failed to prove it. After that we used a factor-analytic approach to find a common version for the three countries. We got a five-factor correlated model in accordance with the theory, but it was reduced from 31 items to 21, and three items moved to other factors. Finally, we provided measurement invariance and reached configural level. To test the narrower facets of factors we used multi-group alignment and found that variances in six parameters differ, mainly in Instability. Despite the difference in the questionnaire items, we proposed a common model for three countries that we called questionnaire IDEA-collectivistic countries (IDEA-CC).

## 1 Emerging adulthood as a developmental stage of modern youth

At the end of the last century, American psychologist Arnett (2000) noticed that the transition to adulthood had changed significantly: young people studied at universities and lived with their parents longer and got married and had children later. According to Arnett (2015), four revolutions (namely, the technological revolution, the sexual revolution, the women’s rights movement, and the youth movement) in the US in the 1960s-70s changed the usual way of taking adult roles. Young people spent more time at universities to successfully socialize, as a result, they started work later and remained financially dependent on their parents.

Arnett interviewed youth and found that they idolized freedom, called their life period as “roleless role,” and avoided taking on adult responsibilities. A lack of commitment allowed youth to spend more time on self-discovery and aspirations, but they became disillusioned with life if their dreams did not come true. Based on these findings, Arnett proposed a new developmental concept – emerging adulthood that covers age from 18 to 29. Arnett (2000) identified five main characteristics – instability, self-focus, identity exploration, experimentation, and the feeling between adolescence and adulthood (Arnett, 2024).

Researchers discussed the opportunities and limitations of the generalizability of the concept. Hendry and Kloep (2007), Côté et al. (2008), and Côté (2014) argued that culture and socioeconomic conditions played a key-role in the transition to adulthood. In high-income countries, parents can support their children financially so they do not have to work, while in low-income countries young people have to earn money as early as they can (Jensen and Arnett, 2014; Katsiaficas, 2017). Furthermore, youth from the Western and Eastern countries want to achieve the same goals, but they have different motives. For instance, financial independence is considered the most important marker of adulthood, but young people from the US strive for achievement, while young people from China want to help their families (Nelson et al., 2004; Nelson and Chen, 2007; Arnett, 2024). Next, the stress levels differ among emerging adults in individualistic and collectivistic countries, and Russian youth have the highest indicators of stress due to occurring world changes (Delvecchio et al., 2023). Finally, youth may gain their self-identity and its development according to cultural expectations (Kagitcibasi, 2017). Hence, we cannot ignore socio-economic and cultural factors because they play a significant role in explaining how to become an adult in the modern world.

This conclusion allows us to consider emerging adulthood not as a universal age stage but a specific trajectory of growing up, along with the traditional one. This means that characteristics of emerging adulthood vary between countries, because four revolutions that initiated changes in the timing of growing up did not affect them with the same intensity (Table 1). The Hofstede’ and Inglehart’s approaches include eight indicators to highlight the difference in cultural values of the countries studied (Haerpfer et al., 2023; Hofstede Insights, 2023). Despite all young people starting to manage their lives earlier to *avoid uncertainty*, youth from the collectivistic countries with an orientation for *distance of power* respect the elderly and get help from them. Along with this, youth from collectivistic countries endorse the value of *restraint* (as opposite to *Indulgence*) which makes them humbler in their wishes, despite all young people being focused on themselves.

**Table 1:** Prevailing values in the countries investigated, compared with the USA.

Country/value	PD	I	MAS	UB	LTO	Indul	S/T	Su/Se
Armenia	85	17	50	88	38	25	T	Su
China	80	43	66	30	77	24	S	Su/Se
Russia	93	46	36	95	58	20	S	Su
United States	40	60	62	46	50	68	S	Se

PD, Power Distance index by Hofstede; I, Individialism index by Hofstede; MAS, motivation toward achievement and success index by Hofstede; UB, uncertainty avoidance index by Hofstede; LTO, long-term orientation index by Hofstede; Indul, indulgence index by Hofstede; S/T, secularism/traditionalism according to Inglehart-Welzel values; Su/Se, survival/self-expression according to Inglehart-Welzel values.

To sum up, five emerging adulthood characteristics may reveal depending on the culture type. In order to investigate whether emerging adulthood phenomenon is culturally universal or not, first of all, we must have a culturally invariant feature measurement tool for evaluating emerging adulthood characteristics.

### 1.1 IDEA as an instrument to measure emerging adulthood

Reifman et al. (2007) proposed an Inventory of the Dimensions of Emerging Adulthood (*IDEA*) to measure emerging adulthood characteristics. *Self-focus* means that young people devote their time to themselves and learn to live independently. *Identity exploration* involves finding themselves in work and relationships and making life choices. *Experimentation* is a close notion to the previous one, and it implies trying something new and finding what suits for them. Lack of consistency leads to *instability*, because young people look for their place in life. Finally, this time is of *feeling in-between*, because youth come across the transition from adolescence to adulthood (Arnett, 2024).

*IDEA* has been adapted in Brazil, China, Greece, Italy, Japan, Spain, Malaysia, Mexico, the Netherlands, Poland, Russia, and Turkey. Factor structures of all these versions differ from the original one, and it is still challenging to compare emerging adulthood characteristics between countries (e.g., Kuang et al., 2023; Zagórska et al., 2023). For example, in the Netherlands, researchers proposed three versions of the questionnaire for local different ethnic groups (Hill et al., 2015). But in Greece (Leontopoulou et al., 2016; Galanaki and Sideridis, 2019), Spain (Fierro and Moreno, 2007; Sánchez-Queija et al., 2020), and Russia (Klement’eva, 2023; Yerofeyeva, 2023) researchers independently conducted the psychometric analysis of IDEA and got different versions. The results of measurement invariance were contradictory: different models were obtained in culturally similar Spain and Mexico (Fierro and Moreno, 2007), but the same version was obtained in culturally different Italy and Japan (Crocetti et al., 2015). The five emerging adulthood characteristics were not always confirmed. For instance, in Greece (Leontopoulou et al., 2016), Turkey (Atak and Çok, 2008), Spain (Pérez et al., 2008), China (Kuang et al., 2023), Malaysia (Wider et al., 2016) the model was reduced to three or four factors.

The concept of emerging adulthood allows us to reveal the phenomenology of growing up, but researchers have found it difficult to verify the factor structure of the questionnaire both within and across countries to compare the characteristics of emerging adulthood. It is not entirely clear whether researchers got contradictory results due to the instability of the phenomenon itself or to the different analytic strategies in the adaptations. Therefore, it seems necessary to conduct comparative cross-cultural studies with similar methodology to draw conclusions about the universalism of emerging adulthood and the stability of its characteristics.

The research questions of our study are:

1. Does *IDEA* have cross-cultural invariance?
2. How do the five emerging adulthood characteristics differ across cultures?

To answer these questions, we examined the structural validity of *IDEA* in a sample of young people from Armenia, China, and Russia that dramatically differ in the prevailing values from the US where the concept of emerging adulthood was discovered (Table 1).

## 2 Materials and methods

### 2.1 Procedure and participants

After receiving the permission from the authors of the questionnaire, we translated *IDEA* into Armenian, Chinese and Russian languages, according to ISPOR requirements (Wild et al., 2005). Then, we made the back-translation and compared it with the English version. The resulting items differed minimally from the original version and retained the intended meaning.

The sample consisted of 868 students from Armenia, China and Russia [Armenia – 283 (51 males/232 females), China – 292 (50/242), Russia – 293 (51/242); total male – 152, total female – 716], aged 18–29 years old (Mean = 21.14, SD = 2.66), who live in million-plus cities and study at university. Respondents participated online in 2023: the survey included questions on gender, age, city of residence, higher education, and *IDEA* questionnaire.

### 2.2 Data analysis

We analyzed the data in the Rstudio program 4.3.2 (R Core Team, 2023) using the following packages: *psych* (Revelle, 2023) for descriptive statistics, parallel analysis, very simple structure and the reliability indicators – Cronbach’s alpha and McDonald’s omega; *lavaan* (Rosseel et al., 2023) for exploratory factor analysis, confirmatory factor analysis, exploratory structural equation modeling; *semTools* (Jorgensen et al., 2022) for measurement invariance; *EGAnet* (Golino, 2023), *sirt* (Robitzsch, 2023), and *ccpsyc* (Karl, 2022) for multi-group alignment analysis; and *semPlot* (Epskamp et al., 2022) for visualizing the results.

## 3 Results

### 3.1 Testing IDEA in each country

First, we assessed the normality of the distribution using descriptive statistics. For all variables we evaluated the skewness and kurtosis values that do not significantly deviate from normality (George and Mallery, 2016). Then we performed confirmatory factor analysis (CFA) with the diagonally weighted least squares (DWLS) estimator to test the fit of the baseline model for each sample (*N*_Armenia_ = 283, *N*_China_ = 292, *N*_Russia_ = 293). We assessed the model fit using the following indices: Chi-sq., CFI, TLI, RMSEA, Robust RMSEA, SRMR. CFI and TLI values exceeding 0.95 indicate good model fit, and values ranging from 0.90 to 0.95 indicate acceptable fit; RMSEA <0.06 and SRMR below 0.08 evaluate the model as having a good fit to the data (Hu and Bentler, 1999). The fit indices showed that the model had acceptable compliance indices for Armenia and China, but low for Russia (Supplementary material 1). Then we tested the original version without the *Other-focused* factor, because it was included to differentiate emerging adulthood from other life stages (Reifman et al., 2007). The fit indices were higher for Armenia and China, but again this model did not fit for Russia (Supplementary material 1).

We also examined the hierarchical and the bi-factor models with the same factor structures (Supplementary material 1). However, all these steps did not advance us to measurement invariance. We then used exploratory structural equation modeling (ESEM)—a more flexible approach that allows all items to load on all factors, providing a more realistic latent structure of the construct (Xiao et al., 2019)—to test the original version and the version without the *Other-focused* factor on the subsamples. We chose the Geomin rotation, which estimates the correlation between factors, as dimensions of *IDEA* are highly correlated (Reifman et al., 2007). These models had the best-fitting indices in the Russian sample, although TLI in all countries was low and СFI was below 0.95, therefore we could not proceed to measurement invariance (Supplementary material 1). Our next step was to find a model using the EFA-CFA procedure on the equal subsamples.

### 3.2 Search for models on a general sample using exploratory factor analysis and their verification using confirmatory factor analysis

We divided the sample into two equal parts: for EFA to analyze the latent factor structure – 434 participants [Armenia – 142 (26 male/116 female), China – 146 (25/121), Russia – 146 (25/121); total male – 76, total female – 358] aged 18 to 29 years old (Mean = 21.09, SD = 2.61); for CFA to test the emerged models – 434 participants [Armenia – 141 (25/116), China – 146 (25/121), Russia – 147 (26/121); total male – 76, total female – 358] aged 18–29 years old (Mean = 21.19, SD = 2.71).

We used parallel bootstrapped analysis (Lim and Jahng, 2019), the Very Simple Structure (VSS) (Revelle, 1979) and Velicer’s minimum average partial (MAP) (Velicer et al., 2000) criterion to determine the number of factors with (31 items) and without (28 items) the *Other-focused* subscale. We applied EFA, not PCA, because EFA extracts the maximum total variance from the variables to show how much each variable contributes to each factor (Alavi et al., 2020). For 31 items, parallel bootstrapped analysis showed seven factors (Supplementary material 2), VSS1 – two, VSS2 – four, MAP – four. For 28 items, parallel bootstrapped analysis showed five factors (Supplementary material 2), VSS1 – two, VSS2 – three, MAP – three.

The questionnaire model is positioned as correlational, so we used two types of oblique rotation – Oblimin and Promax. We chose the minimum residuals estimator, because it makes more accurate estimates about factor loadings (Revelle, 2023). Items that had cross-loadings (multivocal) or the delta between loadings was less than or equal to 0.1 were excluded when assigning items to factors.

As a result, we got 10 unique models, four of which we excluded from further analysis because they included one or two items per latent factor (Supplementary material 3), and the rest we checked using CFA. Although factors are highly correlated with each other, some of them have weak correlations with others - for example, *Feeling in-between* and *Instability* (e.g., Sánchez-Queija et al., 2020; Yerofeyeva, 2023), so we tested the hierarchical and bifactor models as well. We ended up with 18 models, nine of which did not converge (Supplementary material 4). From the rest, we chose six models because they had high fit indices and continued testing them in the measurement invariance analysis (Supplementary material 5).

### 3.3 Measurement invariance analysis

Measurement invariance is a common method in psychology to estimate the factor structure (configural), factor loadings (metric), intercepts (strong) and residual variances (strict) (Putnick and Bornstein, 2016). A higher level of invariance was evaluated according to a decrease in CFI and TLI of more than 0.01, RMSEA of more than 0.015, SRMR less than 0.03. If the values exceed the thresholds, the model obtained at an earlier level of invariance is considered more reliable (Chen, 2007; Morin et al., 2016). To compare the groups by countries, we carried out the analysis on the whole sample (*n* = 868).

#### 3.3.1 Multi-group confirmatory factor analysis (MG CFA)

The five-factor model with oblique Oblimin rotation showed configural invariance, but when we moved to metric invariance, the fit indices deteriorated significantly (Table 2). It explained 45% of the cumulative variance and included 21 items that formed five factors according to the theoretical background. The factor structure slightly changed: items 27 and 28 from the *Identity exploration* scale formed a factor with items 29, 30 from the *Feeling in-between* scale; item 16 from the *Experimentation/Possibilities* scale moved to the *Self-focused* scale.

**Table 2:** The results of measurement analysis.

	χ^2^(df)	CFI	TLI	RMSEA, CI	Robust RMSEA, CI	SRMR
The CFA	514.454 (179)	0.964	0.958	0.047 [0.042–0.050; 0.051–0.059]	0.052 [0.048; 0.056]	0.058
Configural	758.696 (537)	0.979	0.975	0.038 [0.031–0.048; 0.044–0.059]	0.055 [0.050; 0.061]	0.066
Delta 1–0	244.242	0.015	0.017	−0.009		0.008
Metric	980.794 (569)	0.961	0.957	0.050 [0.045–0.049; 0.055–0.060]	0.062 [0.056; 0.068]	0.076
Delta 2–1	222.098	−0.018	−0.018	0.012		0.01

**Figure 1:**
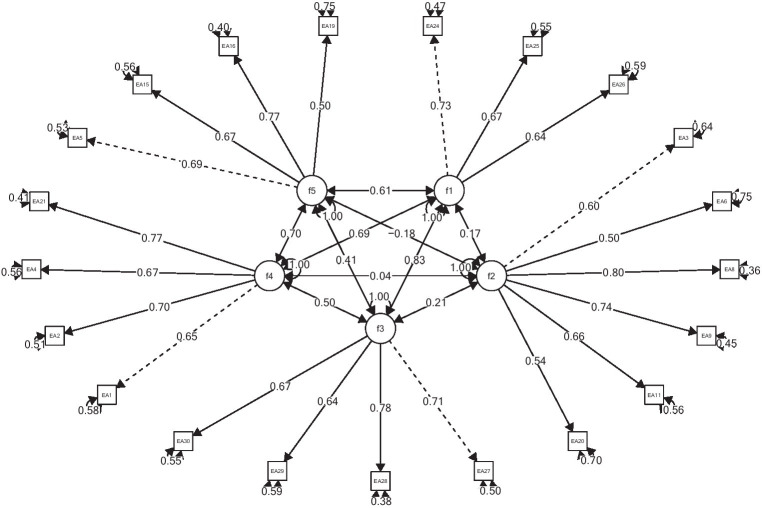
The common model of IDEA in all countries. F1, identity exploration; F2, instability/negativity; F3, feeling in-between; F4, experimentation/possibilities; F5, self-focused.

Item loadings on latent variables range from 0.5 to 0.8; correlations between factors range from 0.17 to 0.83. The weakest correlations are between the factors *Identity Exploration* and *Negativity/Instability* (*r* = 0.17), *Feeling in-between* and *Instability/Negativity* (*r* = 0.21); *Feeling in-between* and *Self-Focused* (*r* = 0.41), and the highest between *Identity Exploration* and *Feeling in-between* (*r* = 0.83), *Self-Focused* and *Experimentation/Possibilities* (*r* = 0.70), *Identity Exploration* and *Experimentation/Possibilities* (*r* = 0.69) (Figure 1).

#### 3.3.2 Multi-group alignment analysis

Next, we performed a Multi-group Alignment Analysis (MGAA) to obtain partially scalar invariance by factor loadings and intercepts for each country and for every subscale. Loadings and intercepts were specified as 0.25 and 0.25, and tolerances set for them were 0.4 and 0.2. *R*^2^ values close to 1 show a higher degree of invariance (Asparouhov and Muthén, 2014; Fischer and Karl, 2019).

We got the following results: loadings = 0.99, intercepts = 1 for *Identity Exploration*; loadings = 0.98, intercepts = 1 for *Instability/Negativity*; loadings = 1, intercepts = 1 for *Feeling in-between*; loadings = 0.99, intercepts = 1 for *Experimentation/Possibilities*; loadings = 1, intercepts = 1 for *Self-focused*. These findings indicate that practically all non-invariance can be explained by group-varying factor means and variances. We found that the intercepts of the groups differed by six items – 3, 6, 8, 20 (*Instability* – 27.8%), 24 (*Identity Exploration* – 11.1%), 15 (*Self-focused* – 8.3%). In the last two cases the percentage of non-invariance did not exceed a cut-off of 25% (Asparouhov and Muthén, 2014), however, for the *Instability/Negativity* subscale this value was higher, so the subscale was non-invariant (Table 3).

**Table 3:** The results of the multi-group alignment analysis.

Subscale/item	Loadings			Percentage of non-invariance item parameters	Intercepts			Percentage of non-invariance item parameters
*Identity Exploration*	Armenia	China	Russia		Armenia	China	Russia	
***EA 24***	0.53	0.53	0.53	0	3.364	3.162*	3.364	11.1%
EA25	0.577	0.577	0.577		3.391	3.391	3.391	
EA26	0.531	0.531	0.531		3.295	3.295	3.295	
*Instability/ Negativity*	Armenia	China	Russia		Armenia	China	Russia	
***EA3***	0.552	0.552	0.552	0	2.853	2.853	2.582*	27.8%
***EA6***	0.487	0.487	0.487		2.209	2.209	1.806*	
***EA8***	0.795	0.795	0.795		2.751	2.453*	2.751	
EA9	0.77	0.77	0.77		2.598	2.598	2.598	
EA11	0.761	0.761	0.761		2.168	2.168	2.168	
***EA20***	0.556	0.556	0.556		2.823*	2.115*	3.090*	
*Feeling in-between*	Armenia	China	Russia		Armenia	China	Russia	
EA27	0.438	0.438	0.438	0	3.315	3.315	3.315	0
EA28	0.518	0.518	0.518		3.495	3.495	3.495	
EA29	0.512	0.512	0.512		3.453	3.453	3.453	
EA30	0.482	0.482	0.482		3.472	3.472	3.472	
*Experimentation/Possibilities*	Armenia	China	Russia		Armenia	China	Russia	
EA1	0.461	0.461	0.461	0	3.332	3.332	3.332	0
EA2	0.52	0.52	0.52		3.478	3.478	3.478	
EA4	0.446	0.446	0.446		3.297	3.297	3.297	
EA21	0.478	0.478	0.478		3.406	3.406	3.406	
*Self-focused*	Armenia	China	Russia		Armenia	China	Russia	
EA5	0.51	0.51	0.51	0	3.134	3.134	3.134	8.3%
***EA15***	0.612	0.612	0.612		3.230	3.522*	3.230	
EA16	0.601	0.601	0.601		3.396	3.396	3.396	
EA19	0.446	0.446	0.446		3.042	3.042	3.042	

*Differences in mean values between countries are shown in bold.

### 3.4 Reliability analysis

We performed the reliability analysis by estimating Cronbach’s alpha and McDonald’s omega (Revelle and Condon, 2019). Standardized Cronbach’s Alpha = 0.84, raw = 0.82, confidence interval 0.82 (0.8; 0.84); McDonald’s hierarchical Omega = 0.63, indicating that there was no general factor; McDonald’s total Omega = 0.9 (reliability indicators for subscale are presented in Supplementary material 6). Reliability indices are high, which allows the modified questionnaire to be used for research purposes.

## 4 Discussion

In this study, we proposed a correlational five-factor model of *IDEA* for collectivistic countries (*IDEA-CC*) that was reduced to 21 items, in which three items moved to other factors compared to the original version. The modified version proved configural invariance in Armenia, China, and Russia. Multi-group alignment analysis showed that six items differ in intercepts that we interpreted based on the Hofstede classification.

The *Instability* scale showed the greatest variability in the countries studied, compared to the other scales. It has lower intercepts for parameters about confusion (EA3) and restriction feelings (EA6) that may be explained by the highest *Power distance* indicator. Young people understand their obligations, and a well-structured society symbolizes the order that helps get answers for questions in uncertain times. The intercept of care (EA20) is the highest in Russia. We suggest that the low *Indulgence* indicator implies tendencies toward adulthood through solving the problems. The intercept indicator of stress (EA8) is also high in Russia and Armenia, but in China it is the lowest that was also found in a recent study (Delvecchio et al., 2023). It is likely that the lowest score of *Independence avoidance* in China can explain how young people cope with unexpected situations due to their cultural worldview. This can either explain why Chinese young people feel less stressed (EA20) due to the transition points. Armenia, like Russia, has a high intercept indicator of worries (EA20) which may be connected with *Traditional culture* in which people become accustomed to cope with problems as their parents and grandparents did.

China has the highest intercept indicator of self-determination (EA24), because from these three countries China is very similar to the US in the value of *Motivation towards achievement and success* and leads in the *Long-term orientation* indicator. It is easier for them to plan their life and understand where they can go if they do certain things. Armenia and Russia have the same intercept of the item measuring independence (EA15) which is higher than China’s indicator. Both these countries are close to each other in the *Power distance* indicator that can relate to people’s perception of adulthood as invulnerability and control that youth want to get.

We proposed a modified reduced version of *IDEA-CC* that can be used in countries with similar indicators on the Hofstede values (Supplementary material 7), which we tested for invariance in three countries. Although we obtained differences explained by cultural features, the factor structure of emerging adulthood characteristics was identical. This model can be used in future cross-cultural studies, but the generalizability of our results should be interpreted with caution that is one of the main limitations of the study. We also think that socioeconomic factors influence the transition to adulthood, but we did not focus on them, and that is also a limitation of the study.

## Data availability statement

The raw data supporting the conclusions of this article will be made available by the authors upon reasonable request.

## Ethics statement

The studies involving humans were approved by the Commission for the Ethical Evaluation of Empirical Research Projects of the Department of Psychology at HSE University. The studies were conducted in accordance with the local legislation and institutional requirements. The participants provided their written informed consent to participate in this study.

## Author contributions

VY: Conceptualization, Data curation, Formal analysis, Investigation, Methodology, Software, Validation, Visualization, Writing – original draft, Writing – review & editing. PW: Data curation, Writing – review & editing. YY: Data curation, Writing – review & editing. AS: Data curation, Writing – review & editing. AG: Data curation, Writing – review & editing. SN-B: Conceptualization, Data curation, Methodology, Project administration, Supervision, Writing – original draft, Writing – review & editing.
