# Supplementary material for: Shimmering emerging adulthood: in search of the invariant IDEA model for collectivistic countries
Source: Front Psychol. 2024 Apr 8;15:1349375. doi: 10.3389/fpsyg.2024.1349375 (PMC11034521; doi:10.3389/fpsyg.2024.1349375)
Supplement: Supplementary file 1 [file Data_Sheet_1.docx]

Supplementary 1. The Results of Testing the Fit of the Baseline Model for Each Sample

|  | χ2(df) | CFI | TLI | RMSEA, CI | Robust  RMSEA, CI | SRMR |
| --- | --- | --- | --- | --- | --- | --- |
| **Armenia** | | | | | | |
| The original version | 917.520 (419) | 0.923 | 0.914 | 0.065  [0.059-0.053; 0.071-0.065] | 0.075  [0.067; 0.082] | 0.089 |
| The original version without *Other-focused* | 748.682 (340) | 0.930 | 0.922 | 0.065  [0.059-0.055; 0.072-0.067] | 0.075  [0.067; 0.083] | 0.088 |
| Hierarchal model | 984.291 (428) | 0.914 | 0.906 | 0.068  [0.062-0.053; 0.074-0.064] | 0.077  [0.069; 0.084] | 0.092 |
| Hierarchal model without *Other-focused* | 815.135 (345) | 0.919 | 0.911 | 0.070  [0.063-0.055; 0.076-0.068] | 0.078  [0.070; 0.086] | 0.092 |
| Bi-factor model | Did not converge | | | | | |
| Bi-factor model without *Other-focused* | 529.820 (322) | 0.964 | 0.958 | 0.048  [0.041-0.045; 0.055-0.059] | 0.062  [0.053; 0.070] | 0.074 |
| ESEM (The original version) | 568.461 (294) | 0.917 | 0.869 | 0.058  [0.050-0.065] |  | 0.035 |
| ESEM (The original version without *Other-focused*) | 527.496 (271) | 0.916 | 0.875 | 0.058  [0.051-0.065] |  | 0.036 |
| **China** | | | | | | |
| The original version | 780.475  (419) | 0.945 | 0.939 | 0.054  [0.048-0.050; 0.060-0.061] | 0.066  [0.059; 0.073] | 0.083 |
| The original version without *Other-focused* | 582.822  (340) | 0.960 | 0.955 | 0.050  [0.043-0.047; 0.056-0.061] | 0.062  [0.055; 0.070] | 0.080 |
| Hierarchal model | 912.843 (428) | 0.927 | 0.921 | 0.062  [0.057-0.052; 0.068-0.064] | 0.072  [0.065; 0.079] | 0.091 |
| Hierarchal model without *Other-focused* | 667.626 (345) | 0.947 | 0.942 | 0.057  [0.050-0.051; 0.063-0.064] | 0.068  [0.060; 0.075] | 0.085 |
| Bi-factor model | Did not converge | | | | | |
| Bi-factor model without *Other-focused* | Did not converge | | | | | |
| ESEM (The original version) | 472.483  (270) | 0.936 | 0.897 | 0.051  [0.043-0.058] |  | 0.033 |
| ESEM (The original version without *Other-focused*) | 548.127  (271) | 0.909 | 0.863 | 0.059  [0.052-0.066] |  | 0.038 |
| **Russia** | | | | | | |
| The original version | 1042.882 (419) | 0.875 | 0.861 | 0.071  [0.066-0.058; 0.077-0.069] | 0.080  [0.073; 0.087] | 0.097 |
| The original version without *Other-focused* | 946.570 (340) | 0.875 | 0.861 | 0.078  [0.072-0.065; 0.084-0.077] | 0.086  [0.079; 0.094] | 0.103 |
| Hierarchal model | 1353.295 (428) | 0.815 | 0.799 | 0.086  [0.081-0.063; 0.091-0.074] | 0.092  [0.085; 0.099] | 0.111 |
| Hierarchal model without *Other-focused* | 1243.715 (345) | 0.815 | 0.797 | 0.094  [0.089-0.072; 0.100-0.084] | 0.101  [0.093; 0.108] | 0.118 |
| Bi-factor model | 955.454 (403) | 0.889 | 0.872 | 0.069  [0.063-0.057; 0.074-0.069] | 0.078  [0.071; 0.085] | 0.093 |
| Bi-factor model without *Other-focused* | 855.927 (322) | 0.890 | 0.871 | 0.075  [0.069-0.066; 0.081-0.078] | 0.085  [0.078; 0.092] | 0.098 |
| ESEM (The original version) | 437.007  (270) | 0.946 | 0.912 | 0.046  [0.038-0.054] |  | 0.032 |
| ESEM (The original version without *Other-focused*) | 505.492  (271) | 0.924 | 0.885 | 0.054  [0.047-0.062] |  | 0.038 |

Supplementary 2. The Results of Parallel Bootstrapped Analysis (for 31 and 28 items respectively)


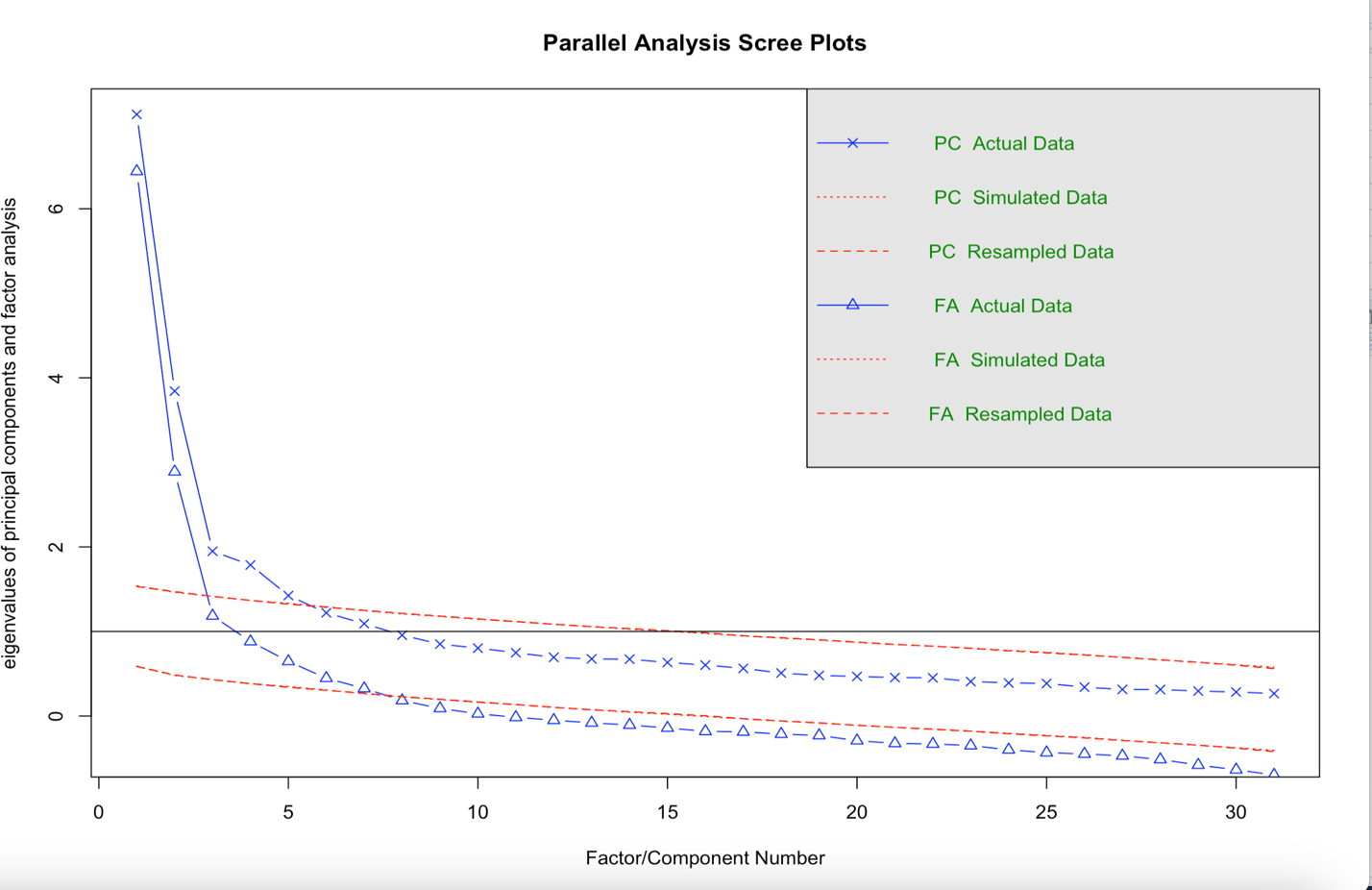


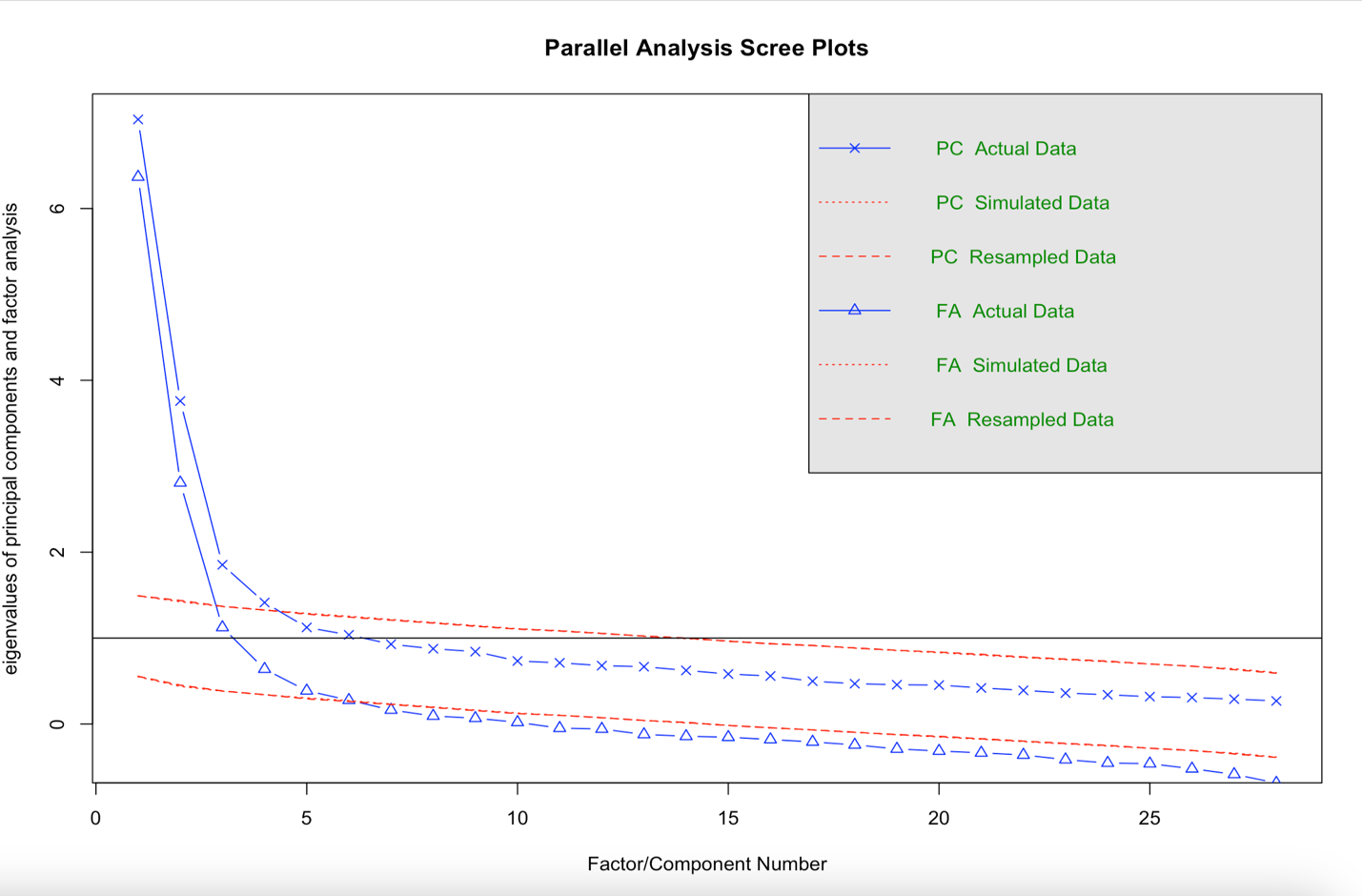


Supplementary 3. The Results of Exploratory Factor Analysis

**31 items, 7 factors – Oblimim (excluded)**

| 1 | 1, 2, 4, 21 |
| --- | --- |
| 2 | 3, 6, 8, 9, 11, 20 |
| 3 | 28, 29, 30 |
| 4 | 14, 18 |
| 5 | 23 |
| 6 | 5, 15, 16, 19 |
| 7 | 12, 24, 25, 26, 27 |
| Multivocal or the delta between loadings was less than or equal to 0.1 | 7, 10, 13, 17, 22, 31 |

**31 items, 7 factors – Promax (excluded)**

| 1 | 1, 2, 4, 21 |
| --- | --- |
| 2 | 3, 6, 8, 9, 11, 20 |
| 3 | 2, 28, 29, 30 |
| 4 | 14, 18 |
| 5 | 13, 23 |
| 6 | 5, 15, 16, 19 |
| 7 | 12, 24, 25, 26, 27 |
| Multivocal or the delta between loadings was less than or equal to 0.1 | 7, 10, 17, 22, 31 |

**31 items, 4 factors – Oblimim (excluded)**

| 1 | 1, 2, 4, 5, 15, 16, 19, 21, 22 |
| --- | --- |
| 2 | 3, 6, 8, 9, 11, 20 |
| 3 | 14, 18 |
| 4 | 26, 27, 28, 29, 30 |
| Multivocal or the delta between loadings was less than or equal to 0.1 | 7, 10, 12, 13, 17, 23, 24, 25, 31 |

**31 items, 4 factors – Promax (excluded)**

| 1 | 1, 2, 4, 5, 15, 16, 17, 19, 21, 22 |
| --- | --- |
| 2 | 3, 6, 8, 9, 11, 20 |
| 3 | 14, 18 |
| 4 | 26, 27, 28, 29, 30 |
| Multivocal or the delta between loadings was less than or equal to 0.1 | 7, 10, 12, 13, 23, 24, 25, 31 |

**31 items, 2 factors – Oblimin**

| 1 | 1, 2, 4, 5, 7, 10, 12, 15, 16, 19, 21, 22, 24, 25, 26, 27, 28, 29, 30 |
| --- | --- |
| 2 | 3, 6, 8, 9, 11, 20, 31 |
| Multivocal or the delta between loadings was less than or equal to 0.1 | 13, 14, 17, 18, 23 |

**31 items, 2 factors – Promax**

| 1 | 1, 2, 4, 5, 7, 10, 12, 15, 16, 19, 21, 22, 24, 25, 26, 27, 28, 29, 30 |
| --- | --- |
| 2 | 3, 6, 8, 9, 11, 20, 31 |
| Multivocal or the delta between loadings was less than or equal to 0.1 | 13, 14, 17, 18, 23 |

**28 items, 2 factors – Oblimin**

| 1 | 1, 2, 4, 5, 7, 12, 15, 16, 19, 21, 22, 24, 25, 26, 27, 28, 29, 30 |
| --- | --- |
| 2 | 3, 6, 8, 9, 11, 20, 31 |
| Multivocal or the delta between loadings was less than or equal to 0.1 | 10, 17, 23 |

**28 items, 2 factors – Promax**

| 1 | 1, 2, 4, 5, 7, 10, 12, 15, 16, 19, 21, 22, 24, 25, 26, 27, 28, 29, 30 |
| --- | --- |
| 2 | 3, 6, 8, 9, 11, 20, 31 |
| Multivocal or the delta between loadings was less than or equal to 0.1 | 17, 23 |

**28 items, 3 factors – Oblimin**

| 1 | 1, 2, 4, 5, 15, 16, 19, 21, 22 |
| --- | --- |
| 2 | 3, 6, 8, 9, 11, 20 |
| 3 | 26, 27, 28, 29, 30 |
| Multivocal or the delta between loadings was less than or equal to 0.1 | 7, 10, 12, 17, 23, 24, 25, 31 |

**28 items, 3 factors – Promax**

| 1 | 1, 2, 4, 5, 15, 16, 19, 21, 22 |
| --- | --- |
| 2 | 3, 6, 8, 9, 11, 20 |
| 3 | 26, 27, 28, 29, 30 |
| Multivocal or the delta between loadings was less than or equal to 0.1 | 7, 10, 12, 17, 23, 24, 25, 31 |

**28 items, 5 factors – Oblimin**

| 1 | 24, 25, 26 |
| --- | --- |
| 2 | 3, 6, 8, 9, 11, 20 |
| 3 | 27, 28, 29, 30 |
| 4 | 1, 2, 4, 21 |
| 5 | 5, 15, 16, 19 |
| Multivocal or the delta between loadings was less than or equal to 0.1 | 7, 10, 12, 17, 22, 23, 31 |

**28 items, 5 factors – Promax**

| 1 | 1, 2, 4, 21 |
| --- | --- |
| 2 | 3, 6, 8, 9, 11, 20 |
| 3 | 7, 27, 28, 29, 30 |
| 4 | 1, 2, 4, 24, 25 |
| 5 | 5, 15, 16, 19 |
| Multivocal or the delta between loadings was less than or equal to 0.1 | 10, 12, 17, 22, 23, 26, 31 |

Supplementary 4. The Results of Confirmatory Factor Analysis

|  | χ2(df) | CFI | TLI | RMSEA, CI | Robust  RMSEA, CI | SRMR |
| --- | --- | --- | --- | --- | --- | --- |
| 31 items, 2 factors  Oblimin/  Promax | 1086.255  (298) | 0.883 | 0.872 | 0.078  [0.073-0.066; 0.083-0.076] | 0.084  [0.078; 0.090] | 0.097 |
| 31 items, 2 factors  Oblimin/  Promax  Hierarchical | Did not converge | | | | | |
| 31 items, 2 factors Oblimin/  Promax  Bi-factor | Did not converge | | | | | |
| 28 items, 2 factors  Oblimin | 883.414  (274) | 0.903 | 0.894 | 0.072  [0.066-0.062; 0.077-0.073] | 0.078  [0.072; 0.084] | 0.092 |
| 28 items, 2 factors  Oblimin  Hierarchical | Did not converge | | | | | |
| 28 items, 2 factors  Oblimin  Bi-factor | Did not converge | | | | | |
| 28 items, 2 factors  Promax | 1086.255  (298) | 0.883 | 0.872 | 0.078  [0.073-0.066; 0.083-0.076] | 0.084  [0.078; 0.090] | 0.097 |
| 28 items, 2 factors  Promax  Hierarchical | Did not converge | | | | | |
| 28 items, 2 factors  Promax  Bi-factor | Did not converge | | | | | |
| 28 items, 3 factors  Oblimin/ Promax | 385.169  (167) | 0.944 | 0.937 | 0.055  [0.048-0.053; 0.062-0.067] | 0.063  [0.056; 0.070] | 0.076 |
| 28 items, 3 factors  Oblimin/ Promax  Hierarchical | Did not converge | | | | | |
| 28 items, 3 factors  Oblimin/ Promax  Bi-factor | 311.948  (150) | 0.959 | 0.948 | 0.050  [0.042-0.056; 0.058-0.070] | 0.060  [0.053; 0.067] | 0.068 |
| 28 items, 5 factors  Oblimin | 292.192  (179) | 0.975 | 0.971 | 0.038  [0.030-0.043; 0.046-0.057] | 0.050  [0.043; 0.057] | 0.062 |
| 28 items, 5 factors  Oblimin  Hierarchical | 534.713  (184) | 0.922 | 0.911 | 0.066  [0.060-0.059; 0.073-0.072] | 0.073  [0.066; 0.080] | 0.083 |
| 28 items, 5 factors  Oblimin  Bi-factor | 442.239  (168) | 0.939 | 0.924 | 0.061  [0.054-0.060; 0.068-0.073] | 0.070  [0.062; 0.070] | 0.076 |
| 28 items, 5 factors  Promax | Did not converge | | | | | |
| 28 items, 5 factors  Promax  Hierarchical | Did not converge | | | | | |
| 28 items, 5 factors  Promax  Bi-factor | 394.072  (165) | 0.948 | 0.934 | 0.057  [0.049-0.059; 0.064-0.073] | 0.066  [0.059; 0.073] | 0.071 |

Supplementary 5. The Results of Multi-group Confirmatory Factor Analysis for Models with High Fit Indices

|  | χ2(df) | CFI | TLI | RMSEA, CI | Robust  RMSEA, CI | SRMR |
| --- | --- | --- | --- | --- | --- | --- |
| 28 items, 3 factors  Oblimin/ Promax | 672.610 (167) | 0.937 | 0.928 | 0.059  [0.054-0.058; 0.064-0.067] | 0.063 [0.058; 0.068] | 0.070 |
| Configural | 920.578 (501) | 0.952 | 0.946 | 0.054  [0.048-0.055; 0.059-0.066] | 0.066 [0.060; 0.072] | 0.078 |
| Metric | 1142.364 (324) | 0.931 | 0.927 | 0.063  [0.058-0.054; 0.068-0.065] | 0.071 [0.065; 0.077] | 0.086 |
| Strong | 1396.140 (569) | 0.906 | 0.906 | 0.071  [0.066-0.061; 0.076-0.071] | 0.078 [0.073; 0.084] | 0.093 |
| Strict | 1572.644 (609) | 0.891 | 0.898 | 0.074  [0.070-0.063; 0.079-0.072] | 0.081 [0.076; 0.087] | 0.102 |

|  | χ2(df) | CFI | TLI | RMSEA, CI | Robust  RMSEA, CI | SRMR |
| --- | --- | --- | --- | --- | --- | --- |
| 28 items, 5 factors  Oblimin | 514.454 (179) | 0.964 | 0.958 | 0.047  [0.042-0.050; 0.051-0.059] | 0.052 [0.048; 0.056] | 0.058 |
| Configural | 758.696 (537) | 0.979 | 0.975 | 0.038  [0.031-0.048; 0.044-0.059] | 0.055 [0.050; 0.061] | 0.066 |
| Metric | 980.794 (569) | 0.961 | 0.957 | 0.050  [0.045-0.049; 0.055-0.060] | 0.062 [0.056; 0.068] | 0.076 |
| Strong | 1238.681 (601) | 0.939 | 0.936 | 0.061  [0.056-0.058; 0.065-0.067] | 0.071 [0.065; 0.076] | 0.083 |
| Strict | 1413.778 (643) | 0.927 | 0.928 | 0.065  [0.060-0.060; 0.069-0.069] | 0.074 [0.069; 0.079] | 0.092 |

|  | Дельта  CFI | Дельта  RMSEA |
| --- | --- | --- |
| Configural |  |  |
| Metric | -0.018 | 0.012 |
| Strong | -0.022 | 0.011 |
| Strict | -0.012 | 0.004 |

|  | χ2(df) | CFI | TLI | RMSEA, CI | Robust  RMSEA, CI | SRMR |
| --- | --- | --- | --- | --- | --- | --- |
| 28 items, 5 factors  Oblimin  Hierarchical | 917.455 (184) | 0.921 | 0.910 | 0.068  [0.064-0.062; 0.072-0.071] | 0.071 [0.066; 0.076] | 0.077 |
| Configural | 1185.260 (552) | 0.940 | 0.931 | 0.063  [0.058-0.060; 0.068-0.070] | 0.073 [0.068; 0.079] | 0.083 |
| Metric | 1778.914 (592) | 0.887 | 0.880 | 0.083  [0.079-0.059; 0.088-0.068] | 0.088 [0.081; 0.095] | 0.101 |
| Strong | 2042.559 (622) | 0.865 | 0.863 | 0.089  [0.085-0.063; 0.093-0.072] | 0.094 [0.087; 0.100] | 0.106 |
| Strict | 2221.040 (664) | 0.852 | 0.859 | 0.090  [0.086-0.064; 0.094-0.073] | 0.095 [0.089; 0.101] | 0.114 |

|  | χ2(df) | CFI | TLI | RMSEA, CI | Robust  RMSEA, CI | SRMR |
| --- | --- | --- | --- | --- | --- | --- |
| 28 items, 5 factors  Oblimin  Bi-factor | 672.989 (168) | 0.946 | 0.932 | 0.059  [0.054-0.058; 0.064-0.067] | 0.063 [0.059; 0.068] | 0.066 |
| Configural | Did not converge | | | | | |

The Models 28 items, 3 factors Oblimin/Promax Bi-factor and 28 items, 5 factors Promax Bi-factor did not converge.

Supplementary 6. The Results of Reliability Analysis for Every Subscale

*Identity Exploration*: standardized Cronbach's Alpha = 0.72, raw = 0.72, confidence interval 0.72 (0.69; 0.75)

*Instability*/*Negativit*y: standardized Cronbach's Alpha = 0.81, raw = 0.81, confidence interval 0.81 (0.78; 0.83)

*Feeling-in-Between*: standardized Cronbach's Alpha = 0.8, raw = 0.8, confidence interval 0.8 (0.78; 0.82)

*Experimentation/Possibilities*: standardized Cronbach's Alpha = 0.79, raw = 0.79, confidence interval 0.79 (0.76; 0.81)

*Self-focused*: standardized Cronbach's Alpha = 0.75, raw = 0.75, confidence interval 0.75 (0.72; 0.78)

Supplementary 7. Questionnaire *IDEA-CC* in four languages – English, Armenian, Chinese and Russian

Instruction

First, please think about this time in your life. By “time in your life,” we are referring to the present time, plus the last few years that have gone by, and the next few years to come, as you see them. In short, you should think about a roughly five-year period, with the present time right in the middle.

For each phrase shown below, please place a check mark in one of the columns to indicate the degree to which you agree or disagree that the phrase describes this time in your life. For example, if you “Somewhat Agree” that this is a “time of exploration,” then on the same line as the phrase, you would put a check mark in the column headed by “Somewhat Agree” (3).

Be sure to put only one check mark per line.

Responses on a Likert scale from 1 to 4 (from Completely   Disagree to Completely Agree)

The English version

| *Identity Exploration* |
| --- |
| 24. time of defining yourself? |
| 25. time of planning for the future? |
| 26. time of seeking a sense of meaning? |
| *Instability*/*Negativit*y |
| 3. time of confusion? |
| 6. time of feeling restricted? |
| 8. time of feeling stressed out? |
| 9. time of instability? |
| 11. time of high pressure? |
| 20. time of many worries? |
| *Feeling-in-Between* |
| 27. time of deciding on your own beliefs and values? |
| 28. time of learning to think for yourself? |
| 29. time of feeling adult in some ways but not others? |
| 30. time of gradually becoming an adult? |
| *Experimentation/Possibilities* |
| 1. time of many possibilities? |
| 2. time of exploration? |
| 4. time of experimentation? |
| 21. time of trying out new things? |
| *Self-focused* |
| 5. time of personal freedom? |
| 15. time of independence? |
| 16. time of open choices? |
| 19. time of self-sufficiency? |

The Armenian version

| *Ինքնության ուսումնասիրում* |
| --- |
| 24. ինքնորոշման ժամանակ է |
| 25. ապագան պլանավորելու ժամանակ է |
| 26. իմաստի որոնման ժամանակ է |
| *Անկայունություն/բացասականություն* |
| 3. շփոթվածության ժամանակ է |
| 6. ժամանակ է, երբ Ձեզ սահմանափակված եք զգում |
| 8. սթրեսային ապրումներ ունենալու ժամանակ է։ |
| 9. անկայունության ժամանակ է |
| 11. ճնշման տակ գտնվելու ժամանակ է։ |
| 20. շատ մտահոգությունների ժամանակ է |
| *Մեջտեղում լինելու զգացողություն* |
| 27. սեփական արժեքների և համոզմունքների մեջ կողմնորոշման ժամանակ է |
| 28. ինքնուրույն մտածել սովորելու ժամանակ է |
| 29. ոչ միշտ, բայց որոշ առումներով ինքդ քեզ մեծահասակ զգալու ժամանակն է |
| 30. աստիճանաբար մեծանալու, հասուն դառնալու ժամանակ է |
| *Փորձարկումներ/հնարավորություններ* |
| 1. շատ հնարավորությունների ժամանակ է |
| 2. որոնումների և ուսումնասիրության ժամանակ է |
| 4. փորձարկումների ժամանակ է |
| 21. նոր բաներ փորձելու ժամանակ է |
| *Ինքնակենտրոնացում* |
| 5. անձնական ազատության ժամանակ է |
| 15. անկախության ժամանակ է |
| 16. ազատ ընտրության ժամանակ է |
| 19. ինքնաբավության ժամանակ է |

The Chinese version

| *同一性探索* |
| --- |
| 24. 定义自己的时期? |
| 25. 规划未来的时期? |
| 26. 寻求意义感的时期? |
| *不稳定性/消极性* |
| 3. 困惑的时期？ |
| 6. 感觉受限的时期? |
| 8. 感到压力很大的时期？ |
| 9. 不稳定的时期？ |
| 1. 高压时期？ |
| 20. 忧虑重重的时期? |
| *不上不下感* |
| 27. 决定你自己的信仰和价值观的时期? |
| 28. 学会独立思考的时期? |
| 29. 在某些方面感觉成熟，但在其他方面感觉不成熟的时期? |
| 30. 逐渐长大成人的时期? |
| *尝试/无限可能性* |
| 1. 有很多可能性的时期? |
| 2. 探索的时期？ |
| 4. 试验的时期？ |
| 21. 尝试新事物的时期? |
| *自我关注* |
| 5. 个人自由的时期? |
| 15. 独立的时期? |
| 16. 开放选择的时期? |
| 19. 自给自足的时期? |

The Russian version

| *Поиск идентичности* |
| --- |
| 24. время самоопределения? |
| 25. время планирования будущего? |
| 26. время поиска смысла? |
| *Нестабильность/Негатив* |
| 3. время замешательства? |
| 6. время, когда вы чувствуете, что вас ограничивают? |
| 8. время, когда вы испытываете стресс? |
| 9. время нестабильности? |
| 11. время большого давления извне? |
| 20. время многих забот? |
| *Ощущение между подростковым возрастом и взрослым* |
| 27. время определиться со своими убеждениями и ценностями? |
| 28. время научиться думать самостоятельно? |
| 29. время почувствовать себя в некотором отношении взрослым, хотя и не всегда? |
| 30. время постепенного взросления? |
| *Эксперименты/Возможности* |
| 1. время многих возможностей? |
| 2. время исследований и поиска? |
| 4. время экспериментирования? |
| 21. время попробовать что-то новое? |
| *Направленность на себя* |
| 5. время личной свободы? |
| 15. время независимости? |
| 16. время свободного выбора? |
| 19. время самодостаточности? |
